# Psychotherapies for generalized anxiety disorder in adults: systematic review and network meta-analysis of randomized-controlled trials

**DOI:** 10.1192/j.eurpsy.2024.155

**Published:** 2024-08-27

**Authors:** D. Papola, C. Miguel Sanz, M. Mazzaglia, P. Franco, F. Tedeschi, S. A. Romero, A. R. Patel, G. Ostuzzi, C. Gastaldon, E. Karyotaki, M. Harrer, M. Purgato, M. Sijbrandij, V. Patel, T. A. Furukawa, P. Cuijpers, C. Barbui

**Affiliations:** ^1^Global health and Social Medicine, Harvard Medical School, Boston, United States; ^2^Neuroscience, Biomedicine and Movement Sciences, University of Verona, Verona, Italy; ^3^Clinical, Neuro- and Developmental Psychology, Section of Clinical Psychology, Vrije Universiteit, Amsterdam, Netherlands; ^4^School of Psychology, Pontificia Universidad Católica de Chile, Santiago, Chile; ^5^Psychology & Digital Mental Health Care, Department of Health Sciences, Technical University Munich, Munich, Germany; ^6^Health Promotion and Human Behavior, Kyoto University Graduate School of Medicine/School of Public Health, Kyoto, Japan

## Abstract

**Introduction:**

Generalized anxiety disorder (GAD) is one of the most common mental disorders in adults. Psychotherapies are among the most recommended treatment choices for GAD, but which should be considered as first-line treatment still needs to be clarified.

**Objectives:**

To examine the most effective and accepted psychotherapy for GAD both in the short and long-term, via a network meta-analysis.

**Methods:**

We searched MEDLINE, Embase, PsycINFO, and the Cochrane Register of Controlled Trials – CENTRAL, from database inception to January 1st, 2023, to find randomized controlled trials (RCTs) of psychotherapies for GAD. Eight psychotherapies (behaviour therapy, cognitive-behaviour therapy, cognitive restructuring, psychoeducation, psychodynamic therapy, relaxation therapy, supportive psychotherapy, and third-wave CBTs) were compared with each other and two control conditions (treatment as usual, waiting list). We followed Cochrane standards when extracting data and assessing data quality and used PRISMA guidelines for the reporting. We conducted random-effects model pairwise and network meta-analyses. We assessed risk of bias of individual studies through the second version of the Cochrane’s Risk of Bias tool and used the Confidence in Network Meta-Analysis (CINeMA) to rate certainty of evidence for meta-analytical results. Severity of GAD symptoms and acceptability of the psychotherapies were our outcomes of interest.

**Results:**

We analysed data from 66 RCTs. Effect size estimates on data from 5,597 participants suggest third wave cognitive-behavioural therapies (standardized mean differences [SMDs] =-0.78; 95%CI=-1.19 to -0.37; certainty=moderate), cognitive-behavioural therapy (CBT) (SMD=-0.68; 95%CI=-1.05 to -0.32 certainty=moderate), and relaxation therapy (SMD=-0.54; 95%CI=-1.04 to -0.05; certainty=low) reduced generalized anxiety symptoms more than treatment as usual (TAU). Relative risks for all-cause discontinuation signalled no differences compared with TAU for all psychotherapies. When excluding studies at high risk of bias, relaxation therapy lost its superiority over TAU. When considering anxiety severity at three to twelve months after completion of the intervention only CBT remained significantly more efficacious than TAU (SMD=-0.58; 95%CI=-0.93 to -0.23).

**Image:**

**Figure:**
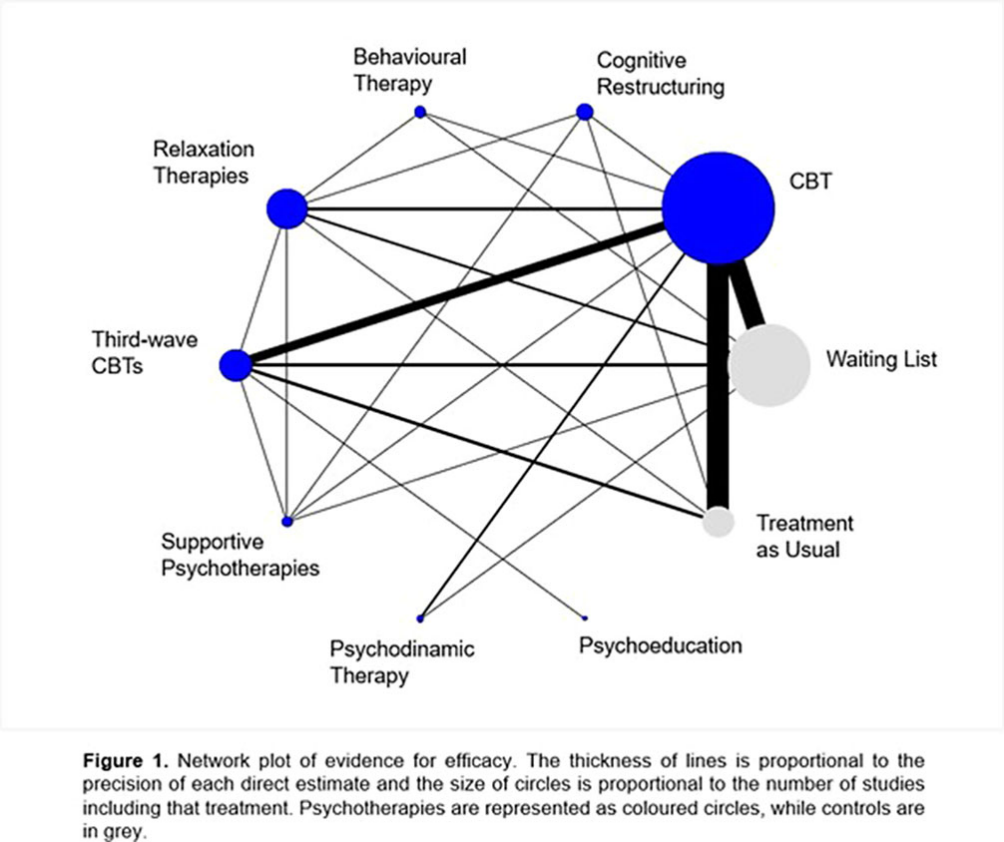

**Conclusions:**

Given the evidence for both acute and long-term efficacy, CBT may represent the reasonable first-line psychological treatment for GAD. Third-wave CBT and relaxation therapy have short-term efficacy and may also be offered. Results from this investigation should inform patients, clinicians, and guidelines. This project is funded by the European Union’s HORIZON EUROPE research programme under grant agreement No 101061648.

**Disclosure of Interest:**

None Declared

